# Supplementary material for: Divergent evolutionary trajectories shape the postmating transcriptional profiles of conspecifically and heterospecifically mated cactophilic Drosophila females
Source: Commun Biol. 2022 Aug 19;5:842. doi: 10.1038/s42003-022-03758-2 (PMC9391497; doi:10.1038/s42003-022-03758-2)
Supplement: Supplementary file 2 — Supplementary Information [file 42003_2022_3758_MOESM2_ESM.pdf]

Supplementary information

Tables

Supplementary Table 1. Library size and mapping statistics. The number of sequenced reads per library and the percentage of unambiguously mapped reads to annotated genes are shown for each LRTs sample in virgins, con- and heterospecifically mated females of *D. mojavensis* and *D. arizonae*.

**Sample**	**Postmating Time**	**Replicate**	***D. mojavensis***		***D. arizonae***	
			Million reads	% Mapping	Million reads	% Mapping
Virgins	na	1	18.5	80.1	23.6	83.0
Virgins	na	2	18.0	79.9	18.7	83.5
Virgins	na	3	16.7	80.3	21.2	83.9
Conspecific	45 min	1	21.0	85.4	22.6	83.5
Conspecific	45 min	2	23.0	86.0	19.1	83.6
Conspecific	45 min	3	21.6	85.4	21.7	84.0
Conspecific	6 hrs	1	25.2	85.6	17.7	83.0
Conspecific	6 hrs	2	24.5	85.4	18.6	84.3
Conspecific	6 hrs	3	21.3	85.5	17.3	83.8
Heterospecific	45 min	1	19.1	84.9	15.8	84.5
Heterospecific	45 min	2	16.6	85.1	16.6	83.8
Heterospecific	45 min	3	17.5	85.7	17.4	85.1
Heterospecific	6 hrs	1	19.4	81.2	21.5	83.2
Heterospecific	6 hrs	2	18.6	79.9	18.1	84.0
Heterospecific	6 hrs	3	15.0	78.2	17.8	83.9

Supplementary Table 2. Number of significant genes. List of significant loci for differential expression (DE), alternative splicing (AS), intron retention (IR), and combinations for *D. mojavensis* and *D. arizonae*. Data for both conspecific and heterospecific matings at two time points are shown.

		**45 min**		**6 hrs**	
		**Conspecific**	**Heterospecific**	**Conspecific**	**Heterospecific**
D. mojavensis	DE only	395	182	315	123
	AS only	433	354	440	0
	IR only	27	21	25	1
	DE + AS	10	2	4	0
	DE + IR	1	0	0	0
	AS + IR	1	0	5	0
	DE + AS + IR	0	0	1	0
	SUM	12	2	10	0
D. arizonae	DE only	34	91	241	285
	AS only	2	28	27	21
	IR only	1	6	4	36
	DE + AS	2	1	3	2
	DE + IR	0	0	0	1
	AS + IR	0	0	0	1
	DE + AS + IR	0	0	0	0
	SUM	2	1	3	4

Figures

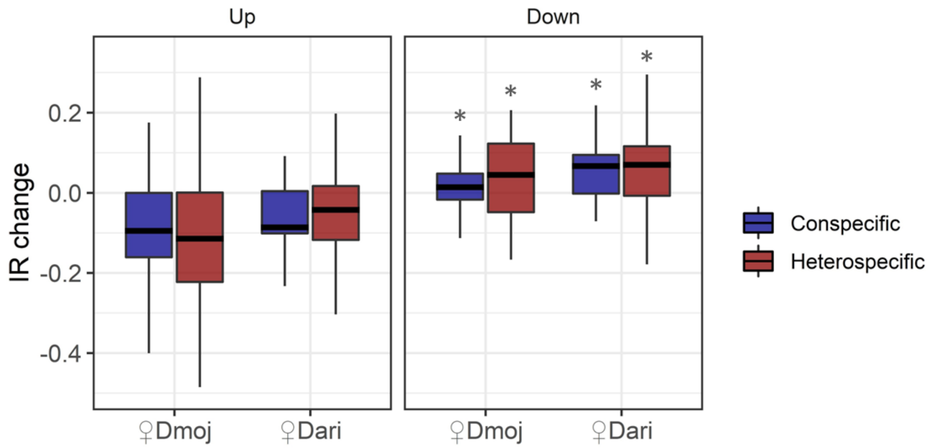

Supplementary Fig. 1 Changes in intron retention rates as estimated for up- vs down-regulated DE genes detected for con- and heterospecific matings between *D. mojavensis* and *D. arizonae*. IR change was estimated as IR mated – IR virgin samples. All mating experiments showed significant increase in IR rates for down-regulated genes with respect to that of up-regulated ones. All significant comparisons with $\alpha = 0.05$ following GLM analysis are indicated with * in the “down” plot. The GLM analysis was performed using categories of up and down regulation as independent variables and the level of IR change as the dependent variable for each mating experiment. GLM analysis was performed after square root transformation while accounting for normal distribution and homoscedasticity of the data. Boxplots represent the median with 25th and 75th percentiles, and whiskers show the 1.5 interquartile range.

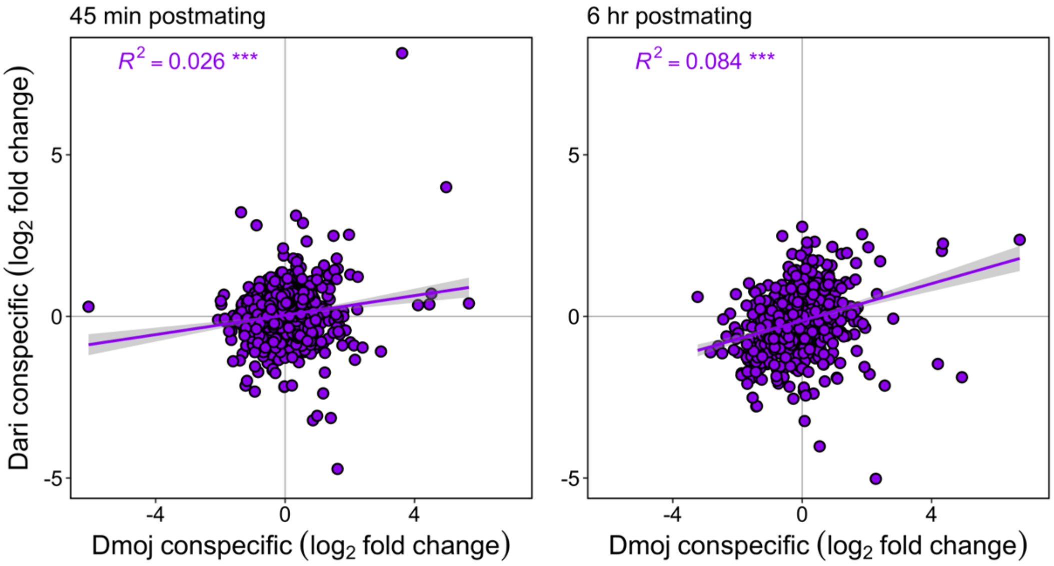

Supplementary Fig. 2 Patterns of transcriptional correlations between non-conspecific-responsive DE genes in *D. mojavensis* vs *D. arizonae*. All comparisons are performed against virgin females. Scatterplots indicate the expression change ($\log_2$) of non-significant DE genes ($FDR_\alpha > 0.05$) for both species at 45 min postmating and 6 hrs postmating. Pearson's R^2 correlation coefficients and linear method trend-lines (with 95% confidence intervals shaded) between the species are indicated. P-values of correlations are noted: *** $P < 0.001$.

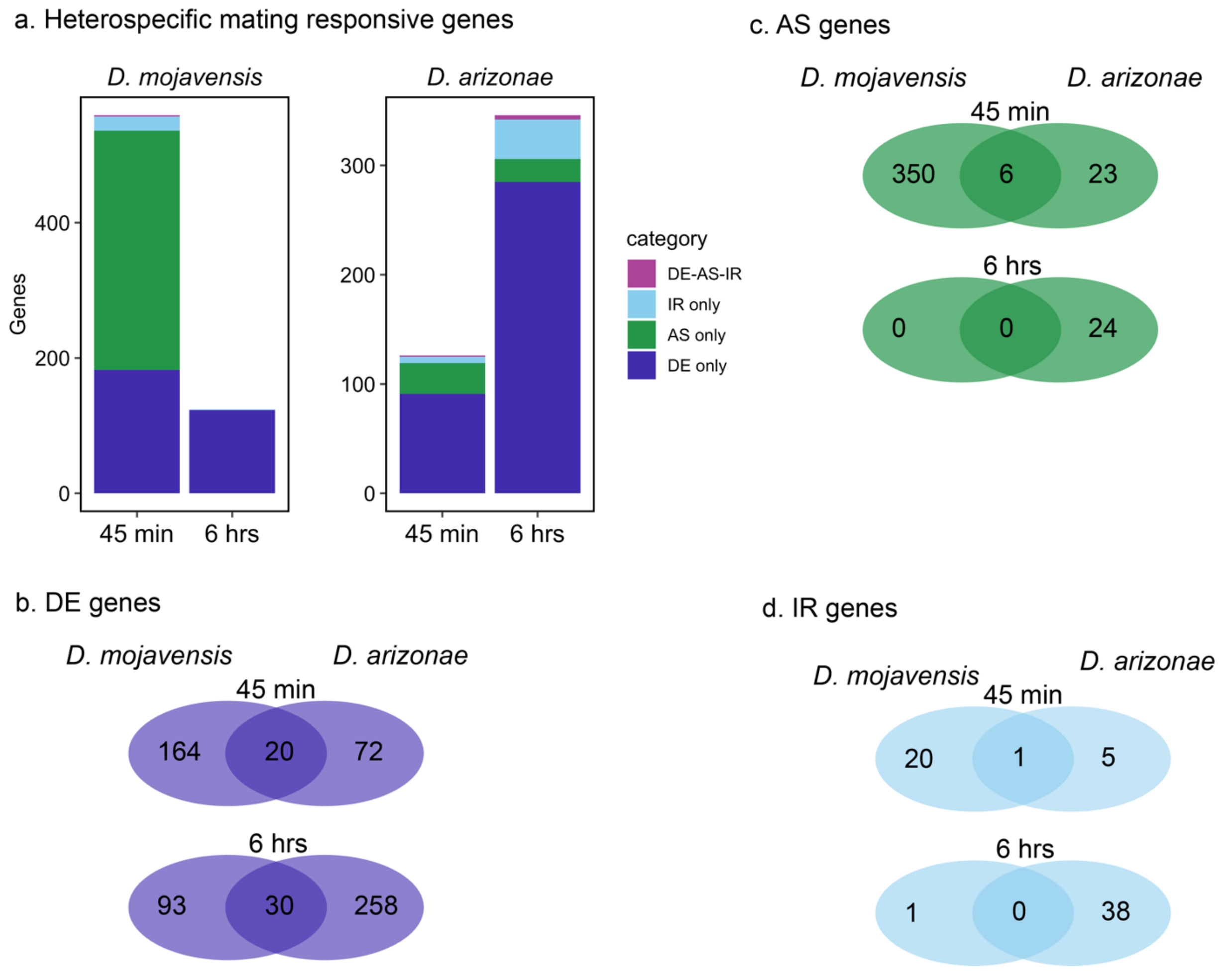

Supplementary Fig. 3 Heterospecific postmating transcriptional response in *D. mojavensis* and *D. arizonae*. a. Number of genes with significant patterns of DE, AS and IR following heterospecific matings in *D. mojavensis* and *D. arizonae*. All comparisons were performed against virgin females at 45 min and 6 hrs postmating ($FDR_{\alpha} = 0.05$). Genes in the DE-AS-IR category, showed significance in two or more of the individual categories (DE, AS and/or IR). **b.** Comparison of **b.** DE genes, **c.** AS genes, and **d.** IR genes from heterospecific matings in each species.
