# Supplementary material for: Divergent evolutionary trajectories shape the postmating transcriptional profiles of conspecifically and heterospecifically mated cactophilic Drosophila females
Source: Commun Biol. 2022 Aug 19;5:842. doi: 10.1038/s42003-022-03758-2 (PMC9391497; doi:10.1038/s42003-022-03758-2)
Supplement: Supplementary file 3 — Description of Additional Supplementary Data [file 42003_2022_3758_MOESM3_ESM.pdf]

Description of Additional Supplementary Files

File name: Supplementary Data 1
Description: The PMPZ isolation source data (fecundity, viability, fertilization, and sperm motility) used for Figure 2.

File name: Supplementary Data 2
Description: The differential expression, alternative splicing, intron retention and rate of molecular evolution (omega) used to generate the remaining graphs.
