# Supplementary material for: Divergent evolutionary trajectories shape the postmating transcriptional profiles of conspecifically and heterospecifically mated cactophilic Drosophila females
Source: Commun Biol. 2022 Aug 19;5:842. doi: 10.1038/s42003-022-03758-2 (PMC9391497; doi:10.1038/s42003-022-03758-2)
Supplement: Supplementary file 6 — Reporting Summary [file 42003_2022_3758_MOESM6_ESM.pdf]

nature portfolio

Corresponding author(s): Luciano Matzkin

Last updated by author(s): 6/14/2022

Reporting Summary

Nature Portfolio wishes to improve the reproducibility of the work that we publish. This form provides structure for consistency and transparency in reporting. For further information on Nature Portfolio policies, see our Editorial Policies and the Editorial Policy Checklist.

Statistics

For all statistical analyses, confirm that the following items are present in the figure legend, table legend, main text, or Methods section.

n/a	Confirmed	
☐	☒	The exact sample size (*n*) for each experimental group/condition, given as a discrete number and unit of measurement
☐	☒	A statement on whether measurements were taken from distinct samples or whether the same sample was measured repeatedly
☐	☒	The statistical test(s) used AND whether they are one- or two-sided<br>*Only common tests should be described solely by name; describe more complex techniques in the Methods section.*
☒	☐	A description of all covariates tested
☐	☒	A description of any assumptions or corrections, such as tests of normality and adjustment for multiple comparisons
☐	☒	A full description of the statistical parameters including central tendency (e.g. means) or other basic estimates (e.g. regression coefficient) AND variation (e.g. standard deviation) or associated estimates of uncertainty (e.g. confidence intervals)
☐	☒	For null hypothesis testing, the test statistic (e.g. *F*, *t*, *r*) with confidence intervals, effect sizes, degrees of freedom and *P* value noted<br>*Give P values as exact values whenever suitable.*
☒	☐	For Bayesian analysis, information on the choice of priors and Markov chain Monte Carlo settings
☒	☐	For hierarchical and complex designs, identification of the appropriate level for tests and full reporting of outcomes
☒	☐	Estimates of effect sizes (e.g. Cohen's *d*, Pearson's *r*), indicating how they were calculated

Our web collection on statistics for biologists contains articles on many of the points above.

Software and code

Policy information about availability of computer code

Data collection	Besides the proprietary software associated with the Illumina reads data collection, no additional software was used in the data collection.
Data analysis	For a number of the analysis the following R packages were used in the manuscript: afex (for GLMM analysis of fecundity, Singmann et al 2021), edgeR (for differential gene expression, Robinson et al 2009), IRFinder (for intron retention analysis, Middleton et al 2017), DESeq2 (differential expression framework used in IRFinder, Love et al 2014), emmeans (used for posthoc testing via Tukey test). Additional analysis sofware included JMP v16, Trimmomatic (Bolger et al 2014), GSNAP (Wu & Nacu 2010), featurecounts (Liao et al 2014), JunctionSeq (Hartley and Mullikin 2016), PAML 4.9 (Yang 2007), MUSCLE 3.8.31 (Edgar 2004), KaKs Calculator 1.2 (Zhang et al 2006).

For manuscripts utilizing custom algorithms or software that are central to the research but not yet described in published literature, software must be made available to editors and reviewers. We strongly encourage code deposition in a community repository (e.g. GitHub). See the Nature Portfolio guidelines for submitting code & software for further information.

Data

Policy information about availability of data

All manuscripts must include a data availability statement. This statement should provide the following information, where applicable:

- Accession codes, unique identifiers, or web links for publicly available datasets
- A description of any restrictions on data availability
- For clinical datasets or third party data, please ensure that the statement adheres to our policy

All reads have been deposited at NCBI under BioProject ID PRJNA777940.

Human research participants

Policy information about studies involving human research participants and Sex and Gender in Research.

Reporting on sex and gender	*Use the terms sex (biological attribute) and gender (shaped by social and cultural circumstances) carefully in order to avoid confusing both terms. Indicate if findings apply to only one sex or gender; describe whether sex and gender were considered in study design whether sex and/or gender was determined based on self-reporting or assigned and methods used. Provide in the source data disaggregated sex and gender data where this information has been collected, and consent has been obtained for sharing of individual-level data; provide overall numbers in this Reporting Summary. Please state if this information has not been collected. Report sex- and gender-based analyses where performed, justify reasons for lack of sex- and gender-based analysis.*
Population characteristics	*Describe the covariate-relevant population characteristics of the human research participants (e.g. age, genotypic information, past and current diagnosis and treatment categories). If you filled out the behavioural & social sciences study design questions and have nothing to add here, write "See above."*
Recruitment	*Describe how participants were recruited. Outline any potential self-selection bias or other biases that may be present and how these are likely to impact results.*
Ethics oversight	*Identify the organization(s) that approved the study protocol.*

Note that full information on the approval of the study protocol must also be provided in the manuscript.

Field-specific reporting

Please select the one below that is the best fit for your research. If you are not sure, read the appropriate sections before making your selection.

☐ Life sciences ☐ Behavioural & social sciences ☒ Ecological, evolutionary & environmental sciences

For a reference copy of the document with all sections, see nature.com/documents/nr-reporting-summary-flat.pdf

Ecological, evolutionary & environmental sciences study design

All studies must disclose on these points even when the disclosure is negative.

Study description	The study focused on the analysis of con- and heterospecific crosses between the sister cactophilic Drosophila species D. mojavensis and D. arizonae. We assessed transcriptional profiles in the lower reproductive tracts of females following the con- and heterospecific matings. Additionally we determine the reproductive consequences (fertility, viability and sperm storage ability) folowing these types of matings.
Research sample	All experiments were carried out using D. mojavensis and D. arizonae isofemale lines originally collected from Anza Borrego Desert State Park, Borrego Springs, CA (in 2002) and Guaymas, Sonora, Mexico (in 2000), respectively.
Sampling strategy	Transcriptional analysis occurred at two time points, 45 minutes and 6 hours post copulation.
Data collection	Sample collection for the transcriptomics was done by Fernando Diaz, Carson Allan, Xingsen Chen and Joshua Coleman. DAPI data collections was done by Jeremy Bono. Fecundity and viability data was collected by Fernando Diaz and Carson Allan. Sperm motility data was collected by Luciano Matzkin.
Timing and spatial scale	All samples for the RNAseq analysis were collected within a period of 3 months in 2020. The fecundity and viability assays were performed in June 2021 and the sperm storage and DAPI staining occurred in between January and April 2021.
Data exclusions	No data excluded
Reproducibility	The RNAseq data had three replicates per sample.

Randomization	Flies for the crosses where randomly chosen from their stock vials to perform the experiments.
Blinding	The RNAseq libraries were generated without knowledge of the sample identities as they were prepared. Additionally, libraries were sequenced in mixed pools in Illumina HiSeq flow cells.

Did the study involve field work? ☐ Yes ☒ No

Reporting for specific materials, systems and methods

We require information from authors about some types of materials, experimental systems and methods used in many studies. Here, indicate whether each material, system or method listed is relevant to your study. If you are not sure if a list item applies to your research, read the appropriate section before selecting a response.

Materials & experimental systems

n/a	Involved in the study
☒	☐ Antibodies
☒	☐ Eukaryotic cell lines
☒	☐ Palaeontology and archaeology
☐	☒ Animals and other organisms
☒	☐ Clinical data
☒	☐ Dual use research of concern

Methods

n/a	Involved in the study
☒	☐ ChIP-seq
☒	☐ Flow cytometry
☒	☐ MRI-based neuroimaging

Animals and other research organisms

Policy information about studies involving animals; ARRIVE guidelines recommended for reporting animal research, and Sex and Gender in Research

Laboratory animals	All experiments were carried out using D. mojavensis and D. arizonae isofemale lines originally collected from Anza Borrego Desert State Park, Borrego Springs, CA (in 2002) and Guaymas, Sonora, Mexico (in 2000), respectively.
Wild animals	*Provide details on animals observed in or captured in the field; report species and age where possible. Describe how animals were caught and transported and what happened to captive animals after the study (if killed, explain why and describe method; if released, say where and when) OR state that the study did not involve wild animals.*
Reporting on sex	*Indicate if findings apply to only one sex; describe whether sex was considered in study design, methods used for assigning sex. Provide data disaggregated for sex where this information has been collected in the source data as appropriate; provide overall numbers in this Reporting Summary. Please state if this information has not been collected. Report sex-based analyses where performed, justify reasons for lack of sex-based analysis.*
Field-collected samples	Samples were collected in 2000 and 2002 from the field.
Ethics oversight	*Identify the organization(s) that approved or provided guidance on the study protocol, OR state that no ethical approval or guidance was required and explain why not.*

Note that full information on the approval of the study protocol must also be provided in the manuscript.
